# Decreased MALL expression negatively impacts colorectal cancer patient survival

**DOI:** 10.18632/oncotarget.8094

**Published:** 2016-03-15

**Authors:** Xiaoliang Wang, Junwei Fan, Fudong Yu, Feifei Cui, Xing Sun, Lin Zhong, Dongwang Yan, Chongzhi Zhou, Guilong Deng, Bin Wang, Xiaosheng Qi, Shuyun Wang, Lei Qu, Biao Deng, Ming Pan, Jian Chen, Yupeng Wang, Guohe Song, Huamei Tang, Zongguang Zhou, Zhihai Peng

**Affiliations:** ^1^ Department of General Surgery, Shanghai First People's Hospital, Medical College, Shanghai Jiao Tong University, Shanghai, China; ^2^ Department of Pathology, Shanghai First People's Hospital, Medical College, Shanghai Jiao Tong University, Shanghai, China; ^3^ Department of Gastrointestinal Surgery, Laboratory of Digestive Surgery of State Key Laboratory of Biotherapy, West China hospital, Sichuan University, Guo Xue Xiang, Chengdu, Sichuan China

**Keywords:** biomarker, colorectal cancer, migration, survival

## Abstract

The aim of the present study was to determine whether MALL expression is associated with colon cancer progression and patient survival. MALL mRNA expression was reduced in the tumor tissues of 70% of the colon cancer patients and 75% of the rectal cancer patients as compared to their normal tissues. MALL protein was also significantly reduced in the tumor tissues of colon cancer patients (*P* < 0.001). Increased LOH and methylation of *MALL* was observed in tumor tissues as compared to normal tissues. Reduced MALL expression was associated with vessin invasion, disease recurrence and metastasis or death (*P* ≤ 0.027). Furthermore, patients with MALL-negative tumors had significantly decreased overall survival (OS) and disease-free survival (DFS) (*P* < 0.008 and *P* < 0.011, respectively). Univariate analysis indicated that MALL expression was significantly associated with OS and DFS. Finally, overexpression of MALL suppressed HCT116 and SW480 cell proliferation and inhibited HCT116 migration. MALL may play a role in colorectal cancer progression as suppression of its expression in tumor tissues negatively impacts colorectal cancer patient survival. Further analyses are required to determine if reduced MALL expression is due to LOH and/or methylation.

## BACKGROUND

Despite advances in treatment and screening along with increased adoption of lifestyle and nutritional changes, colorectal cancer remains a major cause of morbidity and mortality worldwide. It is the second leading cause of cancer-related death in the U.S. [1, 2] and Europe [3]. A similar increased incidence has been noted in China [4]. The 5-year survival rate of patients with superficial cancer (i.e., Duke's staging of colorectal cancer) was 93.2% versus those 6.6% in those with distant metastasis [5]; therefore, early diagnosis can improve colorectal cancer management.

Although colonoscopy remains the gold standard, various biomarkers, including genomic (e.g., genetic and epigenetic markers), miRNA, proteomic, and metabolomic markers, with varying sensitivities and specificities have been identified that could aid in the diagnosis of colorectal cancer [6, 7]. Although many promising screening strategies have been reported, a biomarker of colorectal cancer incidence, development, and prognosis has yet to be identified.

In an effort to identify such a marker or panel of markers, we previously established a long serial analysis of gene expression (SAGE) database that was prepared from colon cancer tissues isolated from Chinese patients [8]. This analysis identified 4300 differentially expressed genes of which 2125 were upregulated and 2175were downregulated in colon cancer [8]. Upregulation of IFITM3 and downregulation of Period 3 (PER3) and phospholipase C epsilon 1 (PLCE1) were subsequently validated, and roles for each in colon cancer development have been proposed [8-10]. In addition, the expression of the T-cell differentiation protein-like, MALL (NM_005434.4), was greatly reduced in carcinoma tissue as compared to normal tissue [8]. MALL is a member of the MyD88 adapter-like (Mal) family of proteins that have role in various cancers [11].

In the present study, the reduced MALL expression was confirmed in colon and rectal cancer and normal tissues using real-time PCR analysis and immunohistochemistry, and the correlation of MALL expression levels with patient survival and clinicopathological characteristics was also evaluated. In addition, the *MALL* gene was analyzed for loss of heterozygosity (LOH) and DNA methylation. Finally, the effects of MALL overexpression on cell proliferation and migration were analyzed. These studies will determine the value of MALL as a possible diagnostic and prognostic biomarker of colon cancer.

## MATERIALS AND METHODS

### Patients and specimens

This study included patients with colon or rectal cancer who underwent radical colectomy at the Shanghai Jiaotong University Affiliated First People's Hospital in Shanghai. Eighty patients, including 40 colon cancer patients and 40 rectal cancer patients, were included for the molecular analyses; 203 additional colon cancer patients were included for the immunochemistry analysis described below.

The normal and cancerous portions of the specimens were snap-frozen in liquid nitrogen and stored at −70°C. The identity of the normal and tumor tissue was confirmed by examination of hematoxylin and eosin (H&E)-stained frozen sections of these specimens independently by two pathologists. Tumor staging was also carried out using the 6th edition of the American Joint Committee on Cancer (AJCC) staging system [12]. In addition, clinical data from all the patients were collected via medical record review. Patient follow-up was performed in accordance with the National Comprehensive Cancer Network Practice Guidelines in colon cancer [13]. Disease-free survival (DFS) and overall survival (OS) rates were defined as the interval from the initial surgery to clinically or radiologically proven recurrence/metastasis and death, respectively. The final follow-up occurred on June 29, 2008 with a median observation time for survivors of 61 months (range 9-89 months). All patients provided informed consent according to a protocol approved by the Institutional Review Board of the Shanghai First People's Hospital.

### Real-time PCR for MALL mRNA expression

MALL mRNA levels were examined in 40 colon and 40 rectal cancer tissues and their normal counterparts by real-time PCR analysis with the SYBR Green RNA PCR kit (Fermentas, Shenzhen, China), according to the manufacturer's instructions and the following primers: MALL sense, 5′- CAGCCTCGTTCTTCGC-3′; MALL antisense, 5′- TTCCGTTTGTCATCCA-3′ (171 bp); actin sense, 5′-ACGTGGACATCCGCAAAGAC-3′; actin antisense, 5′-CAAGAAAGGGTGTAACGCAACTA-3′ (308 bp); 18S RNA sense, 5′- CGATGCTCTTAGCTGAGTGT -3′; and 18S RNA antisense, 5′- GGTCCAAGAATTTCACCTCT -3′ (253 bp). MALL expression was normalized to the expression level of actin or 18S RNA, and relative expression was determined using the ΔΔCt method.

### Evaluation of LOH

LOH analysis was performed in 40 colon and 40 rectal cancer tissues and their normal counterparts by quantitative PCR using the following primers: MALL-1 sense, 5′- GTCAGCGGACGTGACAATTAAG-3′; MALL-1 antisense, 5′- AGGCTTCCCAGGGAGATGTT-3′; MALL-2 sense, 5′- GCTTGCAGGACTGACATGAAC-3′; MALL-2 antisense, 5′- CACCTAAGAGGCAGGTTTCTG-3′; MALL-3 sense, 5′- ACATGCCACGATTGTTTCTG-3′; and MALL-3 antisense, 5′- GAAAGCTGGAGTGGGAACAA-3′. Grossly visible normal and cancerous portions of the specimens were separated by the physicians, and the identity of the normal and tumor tissue was confirmed by examination of hematoxylin and eosin (H&E)-stained sections of these specimens independently by two pathologists to ensure that the tumor samples were not contaminated with healthy cells. Primers were designed with the use of Oligo 6 software (NBI, Plymouth, MN, USA) and were synthesized by Shangai Sangon Biotech (Shanghai, China). Genomic DNA was extracted from frozen samples using a Qiagen DNA extraction kit (Qiagen, Hilden, Germany). The reaction mixture contained 15-μL volumes with 6 μL of genomic DNA (20 ng/μL ), 1.5 μL of primers (10μM each), and 7.5 μL of Sybr Green Mix (SYBR Green RNA PCR kit, Fermentas, Shenzhen, China). Amplification was carried out under the following conditions: 95°C for 10 min, followed by 42 cycles at 95°C for 10 sec, 58°C for 20 sec, and 72°C for 60 sec, followed by 1 cycle of 95°C for 5 sec, 55° for 5 sec and 95°C for 5 sec. The copy numbers were calculated using ΔΔct method and peripheral myelin protein (PMP) as an internal reference.

### Bisulfite sequencing PCR (BSP) for methylation

DNA from six colorectal cancer tissues was used to analyze the methylation status of *MALL* by bisulfite methylation analysis using the Methyl Code Bisulfite Conversion Kit (Invitrogen, Carlsbad, CA, USA) and following the manufacturer's instructions. Two fragments comprising 2000 bp of the promoter region and 800 bp of the first exon were evaluated after amplification using the following primers: 1F5′ TAGTTTTGTGGTTTTGATTTGA 3′; 1R5′ ACAACCAACAAATCCTCAAT 3′ (361 bp); 2F 5′ TGAGGATTTGTTGGTTGTAG 3′; 2R 5′ CCAACTCRAACAAAAAAAA 3′ (270 bp). PCR was performed in a total volume of 50 μL that contained 5 μL of 10×buffer, 2 μL MgCl_2_ (50mM), 1 μL dNTPs (10mM), 4 μL primers (10μM each), 2 μL DNA, 0.2 μL Platinum Taq Hifidility (5U/μL, Invitrogen, and 35.8 μL ddH_2_O. The reactions were subjected to an initial incubation at 95°C for 3 min followed by 40 cycles at 95°C for 30 sec, 53°C for 30 sec, 68°C for 40 sec with a final extension 68°C for10 min. Six PCR products of 2000,1000, 750, 500, 250, and100 bp were obtained for each sample. The PCR products were inserted into pMD 18-T (TaKaRa, Japan), and five clones for each PCR product were sequenced using ABI 3730 sequencer (Applied Biosystems).

### Establishment of stable MALL expressing colorectal cells

To determine the effects of MALL expression on colorectal cell proliferation and migration, the SW620 and HCT116 human colorectal cancer cell lines were purchased from Shanghai Institutes for Biological Sciences and infected with pGC-FU-MALL-FLAG lentiviral expression vector (Genechem) to establish stable MALL-expressing cells as previously described [10]. The inserted sequences were confirmed as NM_005434.4 using the following primers: MALL-Age I sense, GGGTACCGGTCGCCACCATGGCCTCGCCCGAC-3′ and MALL- Nhe I- antisense,5′-TCATCCTTGTAGTCGCTAGCGTGGTAATAGATGCTGAAG - 3′.

### Cell proliferation and migration assays

Cell proliferation was determined using a MTT assay as previously described [10]. Samples were measured at 570 nm at 0, 24, 48, 72, 96, 120, 144, and 168 h, and a cell growth curve was plotted.

For cell migration analysis, 600 μL/well of L-15 cell culture medium containing 20% FBS (Gibco, Carlsbad, CA, USA) was placed into the lower chamber of an 8-μm pore size transwell chamber. Cells in serum-free L-15 medium containing 0.1% bovine serum albumin (BSA) (8×10^4^ cells/200 μL) were added to the top chamber. After 72 h at 37°C, the migrated cells were stained with 0.1% crystal violet for 20 min and were counted under a microscope.

### Immunochemistry for MALL protein expression

The tissues of 203 patients (86 males and 117 females) who had surgery for colon cancer at the Shanghai First People's Hospital Medical Center from January 2001 to December 2003 were analyzed for MALL protein expression. The mean age of the study participants was 65 ± 15 y (range 22-95 y); and 95 patients had lymph node metastases (LNM). Tissue microarrays were made from normal and tumor tissue pairs from each patient. H&E-stained slides were screened for optimal tumor and normal adjacent tissue (at least 2 cm from the tumor) after which the tissue microarray slides were constructed in collaboration with Shanghai Biochip (Shanghai, China). Two cores were collected from each formalin-fixed, paraffin-embedded colon cancer tissue sample and from each normal colonic mucosa sample with a 2.0-mm-diameter punch instrument. Sections were incubated with 1:200 dilution of rabbit anti-human MALL antibody (Abgent, San Diego, CA) overnight at 4°C, and then incubated with goat goat anti-rabbit HRP (Abcam, UK) for 45 min at room temperature. The sections were incubated with DAB (3,30-diaminobenzidine tetrahydrochloride) for 1 min, counterstained with Mayer hematoxylin, dehydrated, and mounted. The negative control was prepared with normal tissue and without anti-MALL antibody incubation.

Immunoreactivity, based on staining intensity and extent of staining, was evaluated independently by two researchers who were blinded to patient outcome. Staining intensity for MALL was scored as 0 (negative), 1 (weak), and 2 (strong). Staining extent was scored as 0 (0%), 1 (1-25%), 2 (26-50%), 3 (51-75%), and 4 (76-100%), depending on the percentage of positively stained cells. The sum of the staining intensity and the staining extent scores was used as the final staining score. The specimens were divided into the following three groups, according to their overall scores: negative (0-1), weakly positive (2-4), and strongly positive (5-6) [14]. Weakly positive and strongly positive were considered as positive.

### Western blot analysis

Whole-cell lysates were prepared from the cells as described previously [15], and the proteins were separated by SDS-PAGE. After transfer of the proteins, the membranes were blocked using 5% non-fat dry milk and incubated with primary antibodies specific for MALL, ERK, or phosphorylated ERK (p-ERK; all from CST, Boston, USA) for overnight at 4ËšC. After the membranes were incubated in the appropriate horse radish peroxidase-conjugated secondary antibodies (CST; 1:5000 dilution) for 2 hours at room temperature, the proteins were detected using an enhanced chemiluminescence system (Amersham Life Sciences, Buckinghamshire, UK) according to the manufacturer's instructions. Analysis of α-tubulin (CST) was used as a loading control. The images were analyzed quantitatively using ImageJ software (http://rsbweb.nih.gov/ij).

### Statistical analysis

All experiments were performed three times independently. MALL expression data were presented by mean and standard deviation (SD). Differences between the cancer and normal tissues observed in colon or rectal cancer patients were compared using paired-t test for the global expression (ΔCT) of MALL. Patients' demographics and clinical characteristics were presented by count and percentage. McNemar's test or Pearson chi-square test was performed to identify the association of MALL expression levels in different tissues. Pearson chi-square test or Fisher's exact test with Yate's correction was performed to identify the association of MALL expression levels with clinical characteristics. Univariate Cox proportional hazards models were used to evaluate the correlation of OS and DFS relative to MALL expression levels and patient characteristics. The results of Cox regression models were summarized by hazard ratio (HR) with 95% confidence interval (CI). Kaplan-Meier curves with log-rank tests represented the cumulative survival proportion for OS and DFS by MALL expression levels. All statistical assessments were two-sided and evaluated at the 0.05 level of significant difference. Statistical analyses were performed with the SPSS software for Windows, version 18.0 (SPSS Inc, Chicago, IL, USA).

## RESULTS

### Reduced MALL expression in rectal and colon cancer tissues

**Figure 1:**
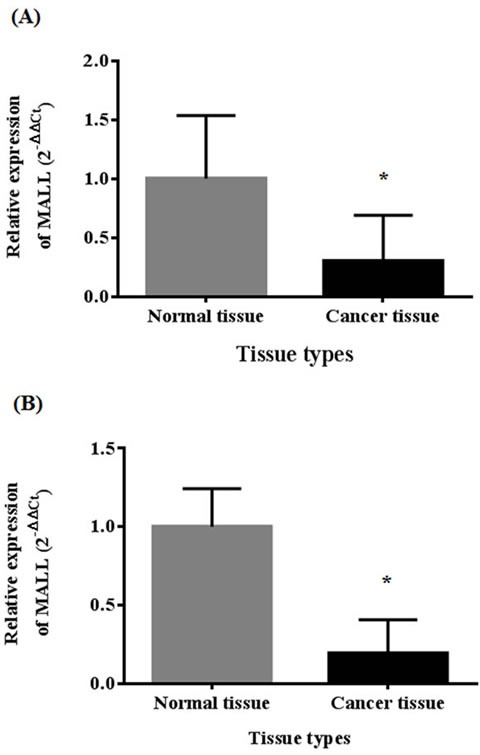
MALL expression in normal and cancer tissues by real-time PCR analysis Analysis of MALL mRNA expression in **A.** 40 colon cancer tissues and **B.** 40 rectal cancer tissues with actin as the internal reference. Data are presented as mean ± standard deviation. *P < 0.05 indicates a significant difference between the normal and cancer tissues.

In a previously established SAGE database, we observed that the *MALL* gene signal was significantly reduced in carcinoma tissue as compared to normal tissue (*P* < 0.001) [8]. In the present study, MALL mRNA expression was confirmed by real-time PCR analysis of normal and tumor tissues isolated from 40 patients with colon cancer and 40 patients with rectal cancer with actin as the internal reference. In patients with colon cancer, the global expression (ΔCT) of MALL was 13.16 ± 1.37 in tumor tissue and 11.44 ± 0.90 in normal tissue (*P* < 0.001, Figure 1A); the relative expression (2^ΔΔCt^) was 0.62 ± 1.08 (range 0.03 - 6.08). In 70% of these patients, MALL tumor expression was significantly lower than in the normal tissues; in 25%, no significant difference between tumor and normal tissues was detected. Similarly, in patients with rectal cancer, the global expression of MALL was 15.54 ± 2.22 in tumor tissue and 13.15 ± 2.04 in normal tissue (*P* < 0.001, Figure 1B), and the relative expression was 2.85 ± 10.80 (range 0.002 - 57.48). Again, 75% of the patients analyzed had significantly reduced expression in tumor tissues; no significant difference between tumor and normal tissues was detected in 15% of patients. These results are consistent with our previous SAGE analysis that shows that MALL was reduced in both colon and rectal tumors [8].

Immunohistochemical analysis of 203 normal and colon cancer pairs revealed that the distribution of MALL expression was significantly different between normal and tumor tissues (*P* < 0.001; Table 1). A significantly greater percentage of tumor tissues were negative for MALL expression as compared to normal tissue (28.6% vs. 7.9%, *P* < 0.001). This was also observed in patients with and without metastasis (31.6% vs. 6.3%, *P* < 0.001 in subjects with metastasis; 25.9% vs. 9.3%, *P* < 0.001 in subjects without metastasis). No association between MALL expression and metastasis was found regardless of tissue types (normal tissue: *P* = 0.437; cancer tissue: *P* = 0.374). Moreover, MALL expression in LNM and cancerous tissue was not statistically different (*P* = 0.109 based on the McNemar's test; Table 1).

**Table 1:** Expression of MALL in normal and colon cancer tissues

Expression of MALL	Normal tissue	Tumor tissue	*P*-value ^†^
All subjects			<0.001*
No. of subjects	203	203	
Negative	16 (7.9)	58 (28.6)	
Positive	187 (92.1)	145 (71.4)	
Subjects with metastasis			<0.001*
No. of subjects	95	95	
Negative	6 (6.3)	30 (31.6)	
Positive	89 (93.7)	65 (68.4)	
Subjects without metastasis			<0.001*
No. of subjects	108	108	
Negative	10 (9.3)	28 (25.9)	
Positive	98 (90.7)	80 (74.1)	
*P*-value ^‡^	0.437	0.374	
	LNM tissue	Tumor tissue	*P*-value ^†^
Subjects with metastasis who provided LNM tissue			0.109
No. of subjects	66	66	
Negative	28 (42.4)	22 (33.3)	
Positive	38 (57.6)	44 (66.7)	

Data are presented as n (%).

Comparison of ^†^ normal and tumor tissues; ^‡^ patients with and without metastasis.

*P*-value are based on the ^†^ McNemar's test for association between MALL expression and tissue types, and on the ^‡^ Person χ^2^ test for association between MALL expression and metastasis.

* *P*<0.05 indicates a significant difference.

### Association of reduced MALL expression in colon cancer with clinicopathologic parameters

**Figure 2:**
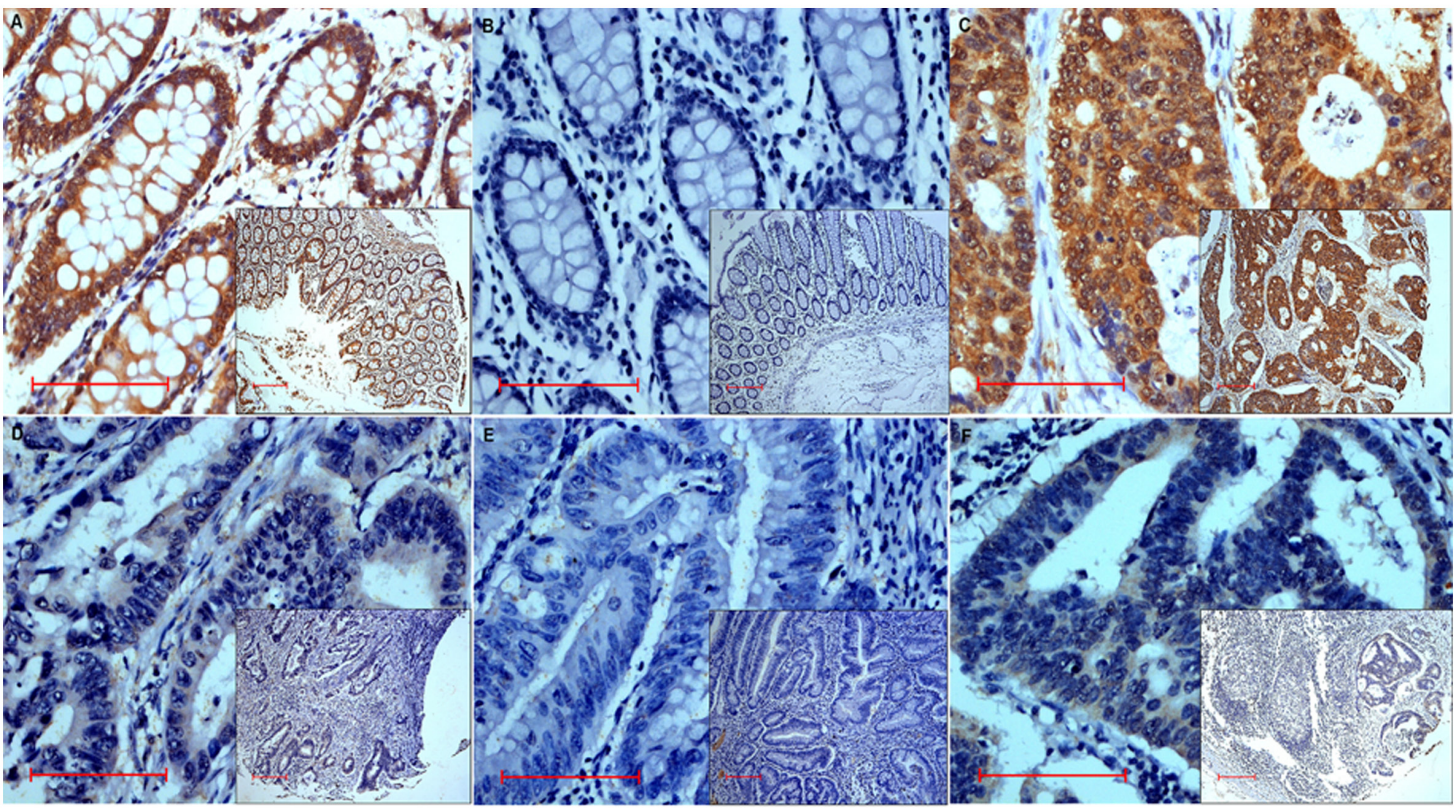
Representative images of MALL immunohistochemistry analysis showing MALL-positive **A.** Normal tissue exhibiting strongly positive expression and **B.** negative control in normal tissue.**C.**-**E.** Tumor tissue exhibiting strongly positive **C.**, weakly positive **D.**, and negative **E.** expression. **F.** Positive expression in metastatic lymph nodes tissue. Bar = 100 μm; original magnification x400 (x100 for insets).

Of the 203 colon cancer patients whose tissues were analyzed by immunohistochemistry, 58 subjects (20 males and 38 females) had MALL-negative tumors (Table 1). No significant differences in age, gender, tumor location, T and M categories, tumor differentiation, and AJCC stage were observed between those patients whose tumors were MALL-positive and MALL-negative (Table 2). However, significant differences in N category, vessin invasion, and recurrence and metastasis were observed between MALL-positive and MALL-negative tumors. Specifically, a large percentage of MALL-negative tumors were classified as N2 as compared to MALL-positive tumors (*P* < 0.008). Moreover, a large proportion of MALL-negative tumors had vessin invasion as compared to MALL-positive tumors (*P* < 0.027). Finally, the incidence of recurrence and metastasis or death was significantly higher in subjects with MALL-negative tumors (*P* = 0.016 and *P* = 0.004, respectively; Table 2). Representative images of MALL-positive normal and tumor tissues are shown in Figure 2.

**Table 2:** Associations between clinical characteristics and MALL expression in colon cancer patients

	MALL expression		
	Negative	Positive	
Variables	(*n* = 58)	(*n* = 145)	*P*-value
Age (y)			0.319
<65	20 (34.5)	61 (42.1)	
≥65	38 (65.5)	84 (57.9)	
Gender			0.151
Male	20 (34.5)	66 (45.5)	
Female	38 (65.5)	79 (54.5)	
Tumor location			0.286
Right colon	30 (51.7)	54 (37.2)	
Transverse colon	5 (8.6)	14 (9.7)	
Left colon	4 (6.9)	16 (11.0)	
Sigmoid colon	19 (32.8)	61 (42.1)	
T category			0.639
T1	2 (3.4)	6 (4.1)	
T2	4 (6.9)	19 (13.1)	
T3	23 (39.7)	53 (36.6)	
T4	29 (50.0)	67 (46.2)	
N category			0.008*
N0	28 (48.3)	80 (55.2)	
N1	13 (22.4)	48 (33.1)	
N2	17 (29.3)	17 (11.7)	
M category			0.118
M0	50 (86.2)	135 (93.1)	
M1	8 (13.8)	10 (6.9)	
Vessin invasion			0.027*
No	50 (86.2)	139 (95.9)	
Yes	8 (13.8)	6 (4.1)	
Differentiation			0.214
Well	29 (50.0)	70 (48.3)	
Moderate	17 (29.3)	57 (39.3)	
Poor	12 (20.7)	18 (12.4)	
AJCC stage			0.351
I-II	27 (46.6)	78 (53.8)	
III-IV	31 (53.4)	67 (46.2)	
Recurrence and metastasis			0.016*
No	25 (46.3)	92 (65.2)	
Yes	29 (53.7)	49 (34.8)	
Patient survival			0.004*
No	30 (51.7)	44 (30.3)	
Yes	28 (48.3)	101 (69.7)	

Data are presented as n (%).

*P*-values are based on the Person χ^2^ test of Fisher's exact test.

* *P*<0.05 indicates a significant difference.

Abbreviation: AJCC, American Joint Committee on Cancer

### Association between MALL expression and colon cancer patient survival

**Figure 3:**
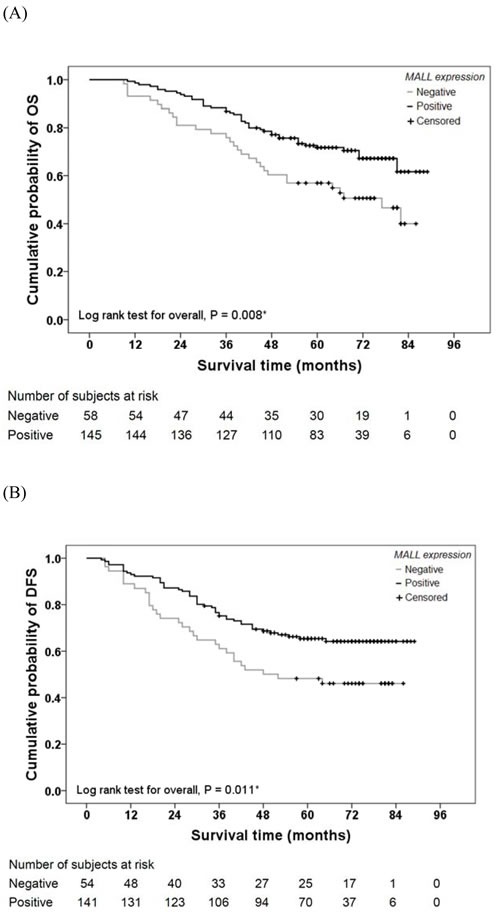
Colon cancer patient survival relative to MALL expression **A.** Overall survival (OS) and **B.** disease-free survival (DFS) of 203 colon cancer patients were analyzed.

To evaluate the possible association between tumor MALL expression and patient survival, Kaplan-Meier curves with a log rank test for OS and DFS were performed (Figure 3). The 1-, 3-, and 5-year OS in subjects with MALL-negative tumors was 93%, 76%, and 52%, respectively; the OS in subjects with MALL-positive tumors was 99%, 88%, and 57%, respectively. The estimated mean OS was significantly different between patients with MALL-positive and MALL-negative tumors (72.8 ± 2.1 vs. 60.1 ± 3.7 months, respectively; *P* = 0.008; Figure 3A). The 1-, 3-, and 5-year DFS in subjects with MALL-negative tumors was 89%, 61%, and 46%, respectively; the DFS in subjects with MALL-positive tumors was 93%, 75%, and 50%, respectively. The estimated mean DFS was significantly different between patients with MALL-positive and MALL-negative tumors (67.9 ± 2.5 vs. 53.8 ± 4.3 months, respectively; *P* = 0.011; Figure 3B).

As shown in Table 3, univariate Cox regression analysis revealed other factors that affect patient survival. In addition to MALL tumor expression, OS and DFS were significantly associated with N category, M category, vessin invasion status, tumor differentiation, and AJCC stage (all *P* ≤ 0.009). DFS was significantly associated with N category, M category, vessin invasion status, tumor differentiation, and AJCC stage (all *P* ≤ 0.002). However, multivariate analysis found no significant association between MALL expression and OS or DFS (Table 4). OS was significantly associated with N category, M category, and tumor differentiation (all *P* ≤ 0.010); DFS was significantly associated with tumor location, M category, and tumor differentiation (all *P* < 0.05; Table 4).

**Table 3:** Association between clinical characteristics and OS and DFS by univariate analysis

	OS		DFS	
Variables	HR (95% CI)	*P*-value	HR (95% CI)	*P*-value
MALL expression				
Positive *vs*. negative	0.54 (0.34, 0.86)	0.009*	0.56 (0.35, 0.88)	0.013*
Age (y)				
≥65 *vs*. <65	0.96 (0.61, 1.53)	0.875	0.98 (0.62, 1.55)	0.938
Gender				
Male *vs*. female	0.75 (0.46, 1.20)	0.223	0.88 (0.56, 1.38)	0.581
Tumor location				
Transverse *vs*. right	0.80 (0.32, 1.93)	0.618	0.83 (0.34, 1.98)	0.669
Left *vs*. right	0.96 (0.42, 2.19)	0.920	0.91 (0.40, 2.08)	0.826
Sigmoid *vs*. right	1.06 (0.64, 1.76)	0.808	1.18 (0.72, 1.92)	0.516
T category				
T2 *vs*. T1	0.30 (0.43, 2.15)	0.232	0.48 (0.81, 2.89)	0.425
T3 *vs*. T1	0.95 (0.22, 4.09)	0.944	1.26 (0.30, 5.34)	0.756
T4 *vs*. T1	2.81 (0.68, 11.55)	0.152	2.96 (0.72, 12.18)	0.132
N category				
N1 *vs*. N0	4.02 (2.18, 7.43)	<0.001*	2.73 (1.57, 4.73)	<0.001*
N2 *vs*. N0	14.07 (7.54, 26.27)	<0.001*	10.22 (5.78, 18.09)	<0.001*
M category				
M1 *vs*. M0	14.74 (8.15, 26.67)	<0.001*	9.93 (4.91, 20.07)	<0.001*
Vessin invasion				
Yes *vs*. no	4.68 (2.55, 8.60)	<0.001*	4.12 (2.16, 7.86)	<0.001*
Differentiation				
Moderate *vs*. well	2.37 (1.34, 4.18)	0.003*	2.26 (1.35, 3.79)	0.002*
Poor *vs*. well	7.50 (4.11, 13.68)	<0.001*	4.87 (2.64, 8.97)	<0.001*
AJCC stage				
III-IV *vs*. I-II	6.66 (3.76, 11.82)	<0.001*	4.24 (2.59, 6.92)	<0.001*

Abbreviations: OS, overall survival; DFS, disease-free survival; HR, hazard ratio; CI, confidence interval; AJCC, American Joint Committee on Cancer

* *P*<0.05 indicates a significant difference.

**Table 4:** Association between clinical characteristics and OS and DFS by multivariate analysis

	OS		DFS	
Variables	HR (95% CI)	*P*-value	HR (95% CI)	*P*-value
MALL expression				
Positive *vs*. negative	0.68 (0.39, 1.18)	0.168	0.58 (0.33, 1.01)	0.055
Age (y)				
≥65 *vs*. <65	1.15 (0.69, 1.91)	0.217	1.02 (0.63, 1.68)	0.927
Gender				
Male *vs*. female	0.73 (0.44, 1.20)	0.731	0.85 (0.53, 1.37)	0.507
Tumor location				
Transverse *vs*. right	0.85 (0.34, 2.18)	0.854	0.94 (0.37, 2.37)	0.896
Left *vs*. right	0.86 (0.35, 2.11)	0.745	0.79 (0.33, 1.86)	0.586
Sigmoid *vs*. right	1.75 (0.99, 3.08)	0.053	1.81 (1.05, 3.13)	0.034*
T category				
T2 *vs*. T1	0.65 (0.09, 4.91)	0.674	0.68 (0.11, 4.28)	0.683
T3 *vs*. T1	0.97 (0.21, 4.45)	0.973	1.12 (0.25, 4.95)	0.884
T4 *vs*. T1	3.12 (0.71, 13.79)	0.133	3.06 (0.71, 13.26)	0.134
N category				
N1 *vs*. N0	3.05 (0.63, 14.83)	0.166	2.42 (0.30, 19.85)	0.411
N2 *vs*. N0	8.34 (1.67, 41.61)	0.010*	6.91 (0.82, 57.85)	0.075
M category				
M1 *vs*. M0	5.79 (2.74, 12.25)	<0.001*	3.69 (1.54, 8.88)	0.003*
Vessin invasion				
Yes *vs*. no	0.58 (0.27, 1.25)	0.164	0.63 (0.29, 1.36)	0.237
Differentiation				
Moderate *vs*. well	1.63 (0.89, 3.00)	0.113	1.75 (1.01, 3.04)	0.045*
Poor *vs*. well	2.97 (1.35, 6.52)	0.007*	2.22 (1.001, 4.91)	0.0496*
AJCC stage				
III-IV *vs*. I-II	1.07 (0.22, 5.27)	0.933	0.94 (0.12, 7.65)	0.955

Abbreviations: OS, overall survival; DFS, disease-free survival; HR, hazard ratio; CI, confidence interval; AJCC, American Joint Committee on Cancer

* *P*<0.05 indicates a significant difference.

### Evaluation of MALL LOH by real-time PCR

**Figure 4:**
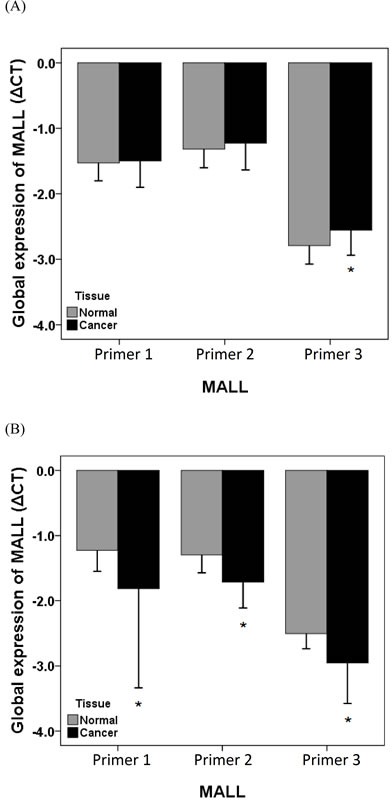
MALL LOH in normal and cancer tissues MALL LOH in **A.** colon and **B.** rectal cancer tissues was determined by real-time PCR using three separate primer sets with PMP as the internal reference. Data are presented as mean ± standard deviation of three independent experiments. *P < 0.05 indicates a significant difference between the normal and cancer tissues.

We next determined if the reduced MALL expression levels were due to LOH. *MALL* LOH was analyzed by real-time PCR analysis of normal and tumor tissues from 40 patients with colon cancer and 40 patients with rectal cancer with three different primer sets as well as PMP as the internal reference. In patients with colon cancer, the global expression (ΔCT) in tumor and normal tissues was −1.50 ± 0.40 vs. −1.53 ± 0.27 (*P* = 0.621) of MALL-1, −1.23 ± 0.41 vs. −1.32 ± 0.29 of MALL-2 (*P* = 0.079), and −2.56 ± 0.38 vs. −2.79 ± 0.28 of MALL-3 (*P* < 0.001), respectively (Figure 4A). The relative expression (2^ΔΔCt^) was 1.02 ± 0.30 (range 0.56 - 1.78) of MALL-1, 0.96 ± 0.22 (range 0.59 - 1.65) of MALL-2, and 0.88 ± 0.25 (range 0.53 - 1.79) of MALL-3. In patients with rectal cancer, the global expression in tumor and normal tissues was −1.81 ± 1.52 vs. −1.23 ± 0.32 (*P* = 0.017) of MALL-1, −1.72 ± 0.41 vs. −1.30 ± 0.28 of MALL-2 (*P* < 0.001), and −2.96 ± 0.62 vs. −2.50 ± 0.23 of MALL-3 (*P* < 0.001), respectively (Figure 4B). The relative expression was 19.71 ± 116.28 (range 0.88 - 736.73) of MALL-1, 1.37 ± 0.35 (range 0.91 - 2.44) of MALL-2, and 1.46 ± 0.45 (range 0.14 - 2.45) of MALL-3.

### Methylation status results

The methylation status of *MALL* was next assessed to determine if this could account for the reduced MALL expression in tumor tissues. As shown in Table 5, several CpG sites in tumor tissues were methylated. In the first fragment, methylation of the overall site was significantly greater in the tumor tissues as compared to that of normal tissues (18.2% vs. 12.4%, *P* = 0.017); similar results were obtained in the second fragment (24.6% vs. 9.5%, *P* < 0.001). In the second fragment, significantly greater methylation at sites 121, 123, 125, 129, 137, 172, 176, 181, 185, 197, 206, 224 was observed in tumor tissues as compared with that in normal tissues (*P* ≤ 0.044).

**Table 5:** Methylation status of each CpG site

	Normal tissue		Cancer tissue		*P*-value
CpG site	Methylated	Unmethylated	Methylated	Unmethylated	
*First fragment*					
70	3	27	4	29	0.789
79	3	27	6	27	0.354
92	4	26	6	27	0.599
135	11	19	20	13	0.058
144	12	18	17	16	0.360
193	2	28	5	28	0.285
221	1	29	2	31	0.612
267	6	24	5	28	0.613
290	3	27	4	29	0.789
302	2	28	1	32	0.498
305	1	29	4	29	0.197
323	1	29	3	30	0.349
329	0	30	2	31	0.171
337	3	27	5	28	0.540
Total	52	368	84	378	0.017*
*Second fragment*					
21	2	28	6	24	0.129
39	4	26	7	23	0.317
48	1	29	2	28	0.554
74	1	29	5	25	0.085
77	0	30	1	29	0.313
81	3	27	7	23	0.166
93	3	27	7	23	0.166
121	1	29	7	23	0.023*
123	1	29	7	23	0.023*
125	1	29	9	21	0.006*
129	1	29	7	23	0.023*
137	2	28	8	22	0.038*
139	2	28	6	24	0.129
148	3	27	7	23	0.166
172	1	29	8	22	0.011*
176	1	29	7	23	0.023*
181	1	29	6	24	0.044*
185	1	29	10	20	0.003*
197	2	28	8	22	0.038*
202	2	28	6	24	0.129
206	2	28	9	21	0.020*
209	2	28	7	23	0.071
217	1	29	4	26	0.161
224	0	30	4	26	0.038*
226	1	29	3	27	0.301
248	9	18	11	19	0.792
263	29	1	30	0	0.313
Total	77	730	199	611	<0.001*

* *P*<0.05 indicates a significant difference.

### Effects of MALL overexpression in colorectal cancer cell lines

**Figure 5:**
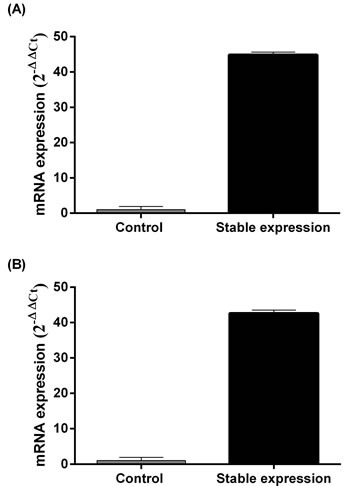
Levels of MALL mRNA expression after its overexpression MALL expression in **A.** SW480 and **B.** HCT116 cell lines was determined by real-time PCR. Data are presented as mean ± standard deviation of three independent experiments.

We initially measured MALL mRNA expression in eight colorectal cancer cell lines, including RKO, HCT116, CW2, HT29, SW480, CACO2, LOVO and SW620, by RT-PCR. HCT116 and SW480 cells had the lowest expression levels of MALL and, therefore, were selected for further analysis of the effects of MALL overexpression *in vitro*, which was confirmed by real-time PCR analysis (Figure 5).

**Figure 6:**
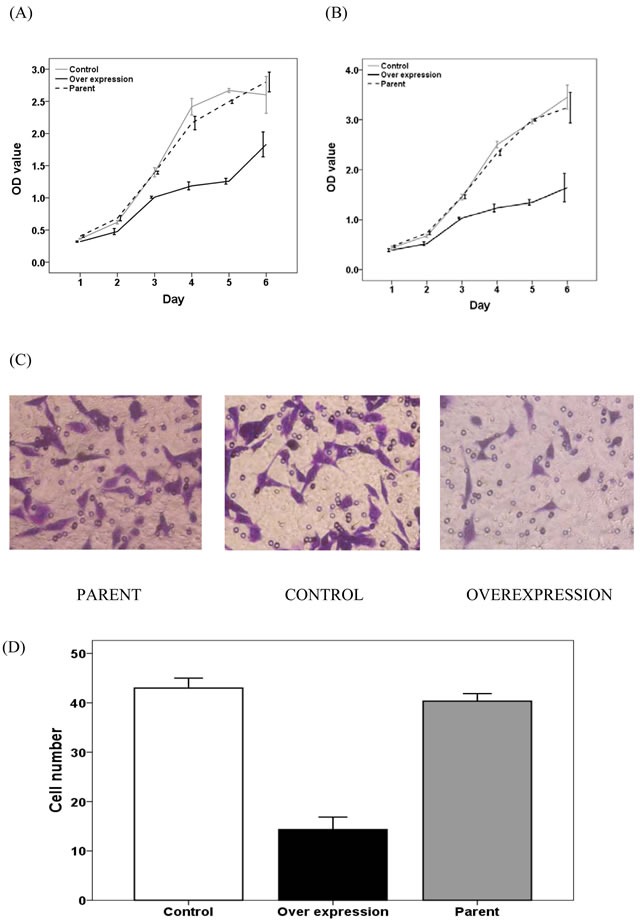
The effect of MALL overexpression on colorectal cell proliferation and migration **A.** SW480 and **B.** HCT116 cell proliferation was determined in control, MALL-overexpressing and parental cells using the MTT assay at the indicate time points. **C.** HCT116 cell migration in control, MALL-overexpressing and parental cells. **D.** Quantitative analysis of the number of migrating cells in C. Data are presented as mean ± standard deviation of three independent experiments.

As shown in Figure 6A and 6B, SW480 and HCT116 cell proliferation was reduced upon MALL overexpression as compared to controls. In addition, HCT116 cell migration was markedly decreased with MALL overexpression as compared with parental and control cells (Figure 6C and 6D).

### MALL suppresses mitogen-activated protein kinase/extracellular signal-regulated kinase (MAPK/ERK) signaling

**Figure 7:**
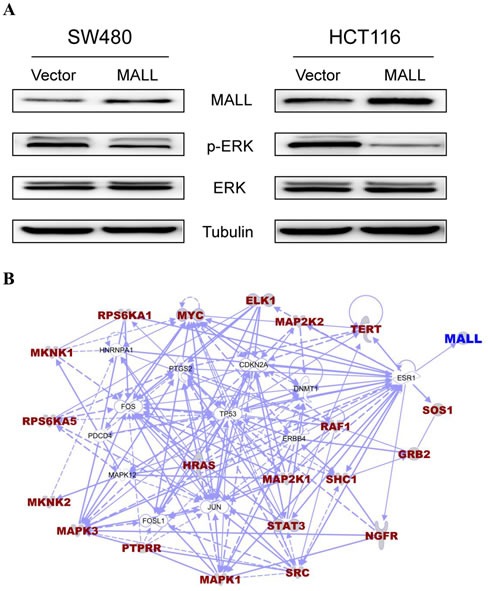
MALL suppresses mitogen-activated protein kinase/extracellular signal-regulated kinase (MAPK/ERK) signaling **A.** p-ERK and total ERK levels were analyzed Western blot analysis in SW480 and HCT116 cells overexpressing MALL or the vector control. **B.** Using the BioCarta database of consensusPathDB, we found genes in the ERK signaling pathway. The interaction between genes in the ERK MAPK signaling pathway and the *MALL* gene were identified using IPA software after removal of unrelated genes.

Because MAPK/ERK signaling pathways are known to drive cell proliferation, survival and metastasis, we analyzed the levels of ERK phosphorylation in MALL-overexpressing SW480 and HCT116 cells to further elucidate the role of MALL in colon cancer cell proliferation and invasion. As shown in Figure 7A, decreased pERK levels were detected in both cell lines overexpressing MALL as compared with the control cells. Densitometry analysis revealed that the reduction was 0.63 ± 0.053 in SW480 cells and 0.28 ± 0.075 in HCT116 cells, respectively.

We next sought to identify genes related to the *MALL* gene and the ERK signaling pathway. Using the BioCarta database (http://pid.nci.nih.gov/) of consensusPathDB (http://consensuspathdb.org/), we first found genes in the ERK signaling pathway. We subsequently used the PhosphoPOINT database [16] to determine if these genes have phosphorylation sites and if they interact with *MALL*. However, our results showed these genes could not interact with the *MALL* gene. In contrast, the interaction between genes in the ERK MAPK signaling pathway and the *MALL* gene (i.e., gene-gene interaction) was identified using Ingenuity Pathway Analysis (IPA) software (http://www.ingenuity.com/products/ipa) after removal of unrelated genes. As shown in Figure 7B, the *MALL* gene may interact with genes in the ERK signaling pathway via the *ESR1* gene, and there might be competitive interaction between them.

## DISCUSSION

We previously found greatly reduced expression of MALL in colon cancer tissues using a SAGE database [8]. To determine whether MALL expression is associated with colon cancer progression and patient survival, we analyzed its expression in colon and rectal cancer tissues and its association with patient clinicopathological characteristics. MALL mRNA and protein expression was reduced in the tumor tissues analyzed, which may be due to LOH and/or methylation of the *MALL* gene. Reduced MALL expression was associated with vessin invasion, disease recurrence and metastasis or death, and patients with MALL-negative tumors had significantly decreased OS and DFS. Finally, overexpression of MALL suppressed HCT116 and SW480 cell proliferation and inhibited HCT116 migration, suggesting that reduced MALL expression contributes to colorectal cancer progression.

MALL is a member of the Mal family of proteins that have role in various tumors [11]. Previous reports have showed the absence of the T cell differentiation protein, MAL, in clear cell carcinoma as well as esophageal carcinoma although it was highly expressed in normal tissue [17, 18]. This suggests a tumor suppressor role for MAL, which was demonstrated by increased tumor cell apoptosis after ectopic expression; suppression of motility and invasion were also noted [18]. In head and neck squamous cell carcinoma, MAL expression was downregulated, and its overexpression reduced cell proliferation, cell cycle progression, and invasion and increased apoptosis [19]. Conversely, other reports have shown MAL overexpression that was associated with T cell lymphoma resistance to therapy [20]. In the present study, MALL expression was greatly reduced in colon and rectal tumor tissues as compared to normal tissues, and ectopic expression inhibited HCT116 and SW480 cell proliferation and HCT116 migration, suggesting that MALL may have a similar tumor suppressor function in colorectal cells.

In the present study, the mechanism by which MALL expression was repressed was explored. Increased LOH and methylation of the *MALL* gene was observed. This is consistent with previous reports in which *MAL* promoter hypermethylation was detected in cervical disease [21, 22] as well as several cancers, including non-small cell lung cancer [23], head and neck squamous cell carcinoma [19], cervical cancer [20, 24], ovarian cancer [25], gastric cancers [26], and colorectal cancer [27]. However, further detailed analyses of the mechanisms of MALL downregulation are necessary to examine whether LOH and methylation are cooperative events or different mechanisms as well as to determine if these events are found in the majority of colon and rectal cancers or only specific subsets.

Epigenetic alterations are commonly observed in a variety of cancers; therefore, analysis of their modifications may represent a biomarker of disease. Determining the methylation status of *MAL* could detect cervical lesions in high-risk human papillomavirus-positive women [28] as well as cervical cancer [20]. Furthermore, *MAL* methylation was associated with platinum sensitivity in epithelial ovarian cancer [25]. Conversely, in gastric cancer, *MAL* promoter hypermethylation was associated with better DFS [26]. In the present study, increased *MALL* methylation was noted in tumor tissues, and MALL-negative tumors were associated with reduced patient survival. Further studies will assess the association of *MALL* methylation status with patient prognosis to determine its value as a prognostic marker.

In the present study, overexpression of MALL suppressed HCT116 and SW480 cell proliferation and inhibited HCT116 migration. Although the precise underlying mechanism by which MALL influences these cellular activities remains unknown, we showed that MALL overexpression in SW480 and HCT116 cells reduced the phosphorylation of ERK possibly through *ESR1*. This suggests that MALL suppresses tumor growth and metastasis through inhibiting the ERK MAPK pathway, which is consistent with previous studies showing that cell adhesion, angiogenesis, invasion, and metastasis in colorectal cancer could be induced by ERK activation. For example, activation of the Ras/Raf/MEK/ERK pathway induces vascular endothelial growth factor (VEGF) expression in human colorectal cancer [29]. Similarly, interactions between the cell-surface urokinase plasminogen activator receptor, an inducer of ERK activation, and integrins are crucial for tumor invasion and metastasis [30]. In addition, the expression of matrix metalloproteinase 7 (MMP7), which is involved in the early stages of intestinal tumorigenesis, is increased by epidermal growth factor (EGF)-induced MAPK signaling [31]. Furthermore, Descot et al. [32] found MAL, a MALL family protein, is the negative regulator of the EGFR-MAPK signaling cascade. In addition to influencing ERK MAPK signaling, MALL may alter actin dynamics and as part of a mechanical feedback system in invading cells [33]. Alternatively, it may influence the expression of genes important for cytoskeletal organization and plasma membrane organization, including s100a4, RhoU, and Krt23, in a manner similar to that described for MAL [34].

The present study is limited due to the absence of *in vivo* analyses. In addition, the mechanism by which MALL influences cell proliferation and migration was not assessed. Given the role of polarity loss in cancer cell metastasis [35], further studies will assess whether MALL influences apical transport in a similar fashion as MAL [36, 37]. Furthermore, because only six patient samples were analyzed in the methylation studies, further analyses are required to fully elucidate the exact mechanism by which MALL was downregulated.

## CONCLUSIONS

Suppression of MALL expression in tumor tissues negatively affects colorectal cancer patient survival. Therefore, MALL may play a role in colorectal cancer progression and may represent a novel therapeutic related and/or diagnostic marker. Further analyses are required to determine if reduced MALL expression is due to LOH and/or methylation.
